# Diverticulum of common hepatic duct leading to obstructive jaundice, a case report

**DOI:** 10.1259/bjrcr.20180105

**Published:** 2019-01-04

**Authors:** Nicola Tarallo, Marco Curti, Valeria Molinelli, Anna Leonardi, Carlo Fugazzola

**Affiliations:** 1 Department of Diagnostic and Interventional Radiology, University of Insubria, Varese, Italy

## Abstract

Choledochal cyst is a dilation of the intrahepatic and/or extrahepatic biliary tree. The pathogenesis is unknown and potentially is multifactorial. In 1977, Todani classified the cysts under five different types according to their morphology, number and distribution along the biliary tree. Presenting symptoms of Choledocal cysts which include upper abdominal pain, acute cholangitis and jaundice, although often they are clinically silent and discovered as an incidental finding. Biliary complications include cholangitis, biliary stones, pancreatitis, portal hypertension and cholangiocarcinoma. We describe a case of a rare Type II Todani cyst located on the right side of the common hepatic duct characterised by a clinical presentation similar to that observed in Mirizzi Syndrome. The treatment of a Type II choledochal cyst consists in cystic excision.

## Introduction

Choledochal cyst (CC) is a dilation of the intrahepatic and/or extrahepatic biliary tree. The pathogenesis is unknown and is potentially multifactorial. CC may be congenital or acquired; 60% of all cases are diagnosed in the first decade of life.^1^ The acquired type has a strong association with abnormal pancreaticobiliary junction (APBJ), resulting in approximately 70% of all cases.^2^ The incidence is higher in Eastern countries, notably Koreas and Japan (1:1000), whereas in Western countries it is 1:100.000–150.000.^3^ Additionally, this rare disease has a female predominance (M/F 1:3).^4^

Choledochal cysts may be found in different clinical scenarios; jaundice, acute cholangitis and abdominal pain or remain asymptomatic. Biliary complications include cholangitis, biliary stones, pancreatitis, portal hypertension and cholangiocarcinoma^5^; however, the tumorigenic process has not been clarified yet.^6^ Todani et al proposed a classification that encompasses five types of CC and have gained widespread acceptance. Type II CC, a diverticulum of the common bile duct (CBD), is the rarest type.^7^

## Case presentation

**Figure 1.**
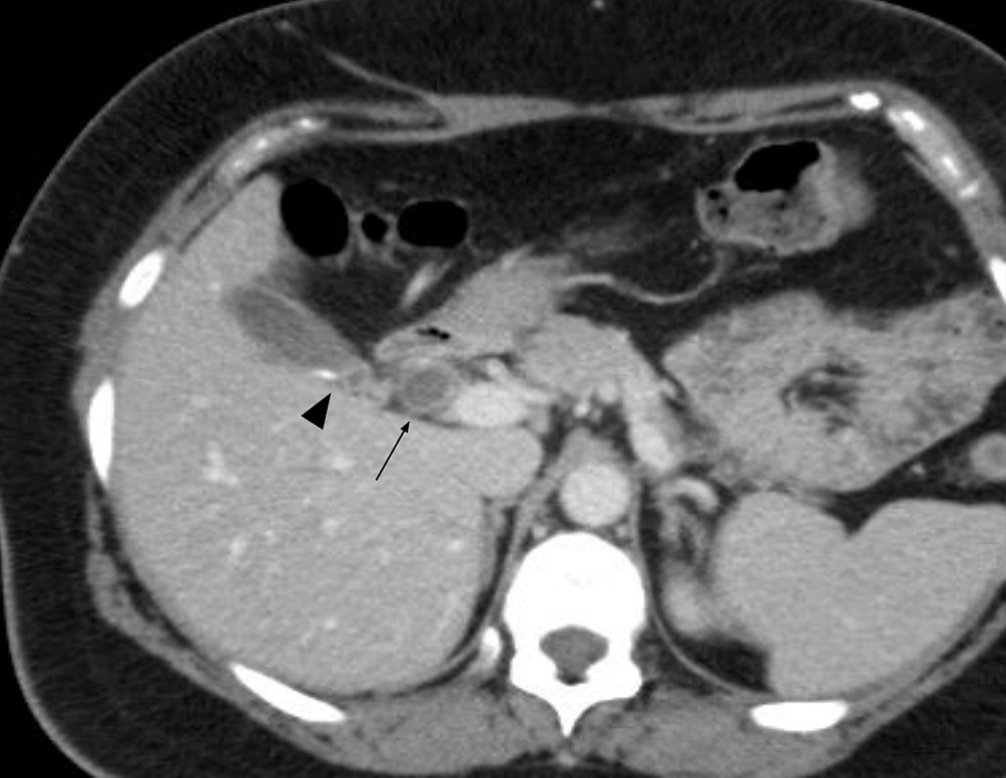
Choledocal diverticulum (Type II Todani class). MDCT venous phase. Biliary stone (arrowhead) in the gallbladder lumen. Diverticulum (arrow), containing biliary sludge, is located on the right side of the CHD. CHD,common hepatic duct; MDCT, multidetector CT.

**Figure 2.**
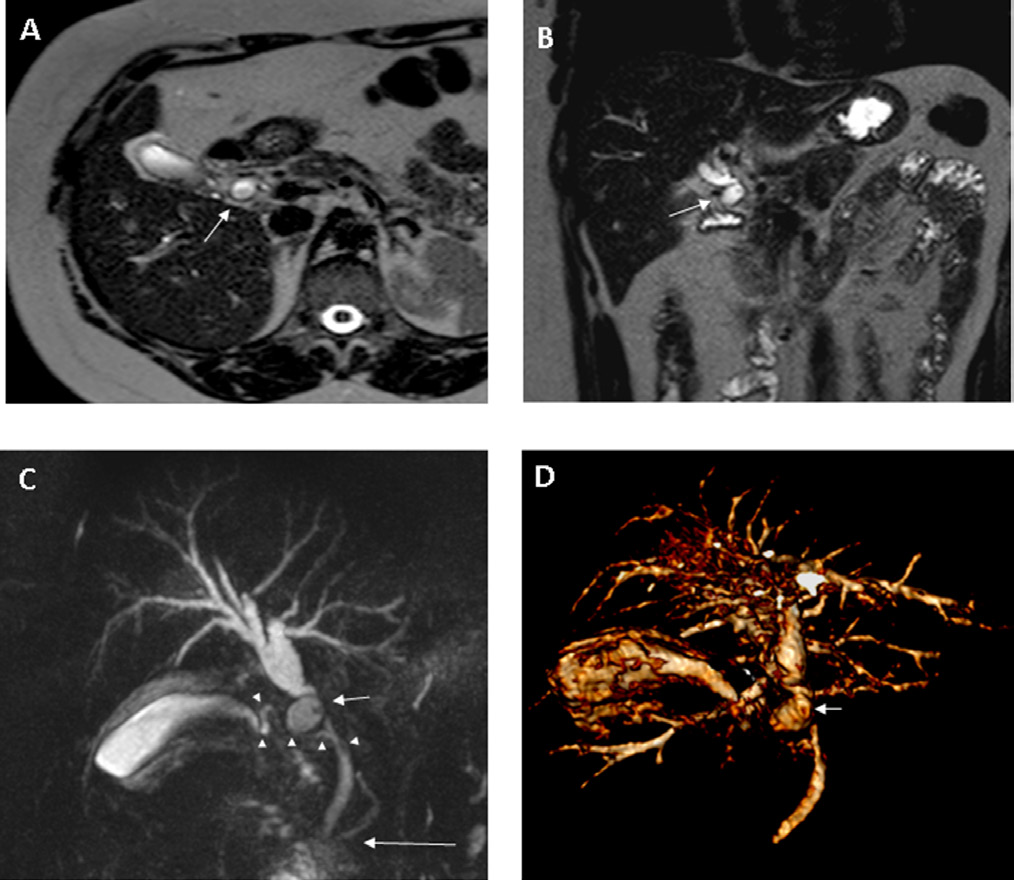
Choledocal diverticulum (Type II Todani class). (A, B) Axial and coronal *T*_2_W TSE images. (C, D) MRCP MIP and VR reconstructions. Diverticulum (short arrow), containing biliary sludge, is located on the right side-of the CHD (A–D); the diverticulum impinges the distal tract of CHD, causing dilation of the biliary system upstream (B, C, D). CBD has a normal diameter (C, D). Cystic duct shows a spiral course and a medial insertion on the CBD (white arrowheads: C); normal pancreatico-biliary junction is detectable (long arrow: C). CHD,common hepatic duct; MDCT, multidetector CT; MIP, maximum intensity projection; MRCP, magnetic resonance cholangiopancreatography; TSE, turbo spin echo.

We describe a case of common hepatic duct (CHD) diverticulum. The patient was a 47-year-old female, characterised by a previous history of neuroendocrine colonic tumour, treated with a right hemicolectomy. She was admitted to the ER unit, complaining of relapsing and remitting abdominal pain, vomiting and jaundice, but with no fever or Murphy’s signs. Lab tests showed incremented bilirubin: 4 mg dl^−1^ (dir 3.6 mg dl^−1^) and AST 176 ALT 197. Firstly, at abdominal ultrasonography, the CHD as well as the intrahepatic biliary ducts were dilated, while the CBD appeared normal. Moreover, a biliary stone was present in the gallbladder lumen. CT confirmed the ultrasonography findings and showed a suspected focal dilation of the biliary tree, cranially to the cystic duct opening (Figure 1). To better study the biliary tree, MRI was performed and revealed a saccular dilation of 15 mm in diameter, located on the right side of the CHD, containing biliary sludge (Figure 2); the diverticulum compressed the distal CHD causing dilation of the biliary tree upstream. The CBD had a normal diameter; the cystic duct showed a spiral course with a medial insertion into the CBD. Therefore, was it concluded that jaundice was caused by the extrinsic pressure of the diverticulum on the distal tract of CHD. In order to preserve the CHD, an unsuccessful attempt to cyst excision was sought. Therefore, it was decided to perform a cholecystectomy and a Roux-en-Y biliodigestive anastomosis according to Hepp-Couinaud.

## Discussion

Choledochal cyst is a rare disorder of the intrahepatic and/or extrahepatic biliary tree and and represents nearly 1% of all benign biliary disorders.^8^ Todani classified these cysts under five different types according to their morphology, number and distribution along the biliary tree.^7,9^ In 1991, a sixth type, isolated cysts dilation of the cystic duct, was included to the Todani classification.^10^ Subsequently, “form fruste” CCs with APBJ and no bile duct dilation were reported.^11^

The distribution of different CC types is not even; Type I represents 50–80% of cases, Type IV 15–35%, Type V 20% and Type III 4%.^11,12^ Type II cyst, present in only 2% of all CCs, is the rarest type and usually origins from the CBD^8^ ; however, its true incidence is difficult to determine, because it is often asymptomatic.^13^ Few cases of Type II cysts above the cystic duct opening have been studied by imaging techniques and are described in either cases reports^13–15^ or pictorial essays.^6,16^

The pathogenesis of CC has always been a hot debate with three different theories mainly quoted. Babbitt hypothesised that every bile duct malformation derives from an APBJ.^17^ The reflux of pancreatic secretion into the biliary tree causes inflammation and deterioration of the biliary duct wall, leading to the formation of a dilation”. Moreover, the increase of pressure could further expand areas of low resistances. However, this theory supports only the formation of Type I or Type IV CCs due to the fact that APBJ is found only in 50–80% of all CC cases. Besides, CCs are less common than APBJ, so their development is thought to be multifactorial.^18^ Other authors define CCs as pure congenital abnormalities, caused by an embryologic overproduction of epithelial cells. Davenport and Basu^19^ noted that neonatal round CCs were characterised by few neurons and ganglions. This lack of neurological stimulation causes a distal obstruction, which could explain the development of CCs. This mechanism was compared to Hirschprung's disease. Finally, Singham^11^ states that all neonatal CCs are round whereas those associated with biliary atresia are fusiform: this statement raised an interesting point of discussion on whether round cysts are congenital and fusiform are acquired.

However, the mentioned theories do not fully explain the development of Type II CCs, which are defined as a true diverticulum of the biliary tree, usually without APBJ^6^; in fact, this association is exceptional.^20^

As mentioned earlier, CCs are often asymptomatic; the triad of jaundice, right upper quadrant pain and a palpable mass is a classical finding in pediatric patients.^21^ Adults that do show symptoms are usually presenting with a clinical history of abdominal pain and vomiting; of these patients, approximately 60–80% of these patients can experience biliary stones, cholangitis, pancreatitis, liver abscess, biliary cirrhosis.^8^ Once diagnosed, CCs must be surgically treated; in fact, these malformations are considered a premalignant state. The overall risk of cancer development is close to 11% and increases with age.^8,22^ Moreover, the carcinogenic evolution in CCs changes according to the type of biliary dilation: Type I–70%, Type IV–20%, Type II–5% and Type III–2%.^5,8^

A correct diagnosis by imaging modalities is crucial. Ultrasonography is the first screening tool for studying biliary tree, mostly in children. CT allows for the better evaluation of the cause of biliary duct dilation. However, for precise assessment of the biliary tree, ultrasonography and CT are not enough and cholangiography is mandatory.^6,8^

Endoscopic retrograde cholangiopancreatography and percutaneous transhepatic cholangiography are the most sensitive techniques; unfortunately, both are invasive and operator-dependent.^6,8,9^ Nowadays, MRI is considered the gold-standard for initial evaluation and diagnosis of CCs; in fact, it is able to accurately assess the intra- and extrahepatic biliary tree, the pancreatic-biliary junction, and to look for associated complications. In literature, it is reported that Magnetic resonance cholangiopancreatography (MRCP) has a 96–100% detection rate for CCs, a 53–100% for diagnosing anomalous APBJ, a 100% for choledocholithiasis, and a 87% for cholangiocarcinomas with concurrent CCs. These excellent results led to consider MRCP the test of choice for pre-operative evaluation.^23^ Furthermore, hepatobiliary MR contrast agents—because of their elimination through the biliary system—can be used for contrast-enhanced MRCP in difficult cases to evaluate the communication between the cystic lesion and the biliary tree.^8^

In our case, MRCP showed the diverticulum located on the right side of the CHD, as well as its compression on the distal CHD: this finding, similar to that observed in Mirizzi Syndrome,^24^ caused obstructive jaundice. Moreover, MR contrast agent—during the hepato-biliary phase—did not fill the diverticulum which is probably due to dense bile inside the cystic neck.

Cancerogenic potential has become a crucial issue in the surgical managing of CCs. In particular for the treatment of Type II CC, and despite their low tendency to evolve to cholangiocarcinoma, cyst excision is commonly used as a surgical approach. However, in cases where the cyst’s neck is wider or APBJ is present, cystic excision with Roux-en-Y cystojejunostomy is mandatory.^13,25^

## Conclusion

Type II CCs are extremely rare, in particular those arising from the CHD. MRCP is the current “gold-standard” imaging modality for initial evaluation and diagnosis of this type of CC. Total cystic excision is the treatment of choice in order to prevent malignant transformation.

## Learning points

- Choledochal cyst is a rare disorder of the intrahepatic and/or extrahepatic biliary tree and represents nearly 1% of all benign biliary disorders.
- Choledochal cysts may be found in different clinical scenarios (jaundice, acute cholangitis, abdominal pain) or may be asymptomatic.
- Type II cyst, present in only 2% of all CCs, is the rarest type and usually origins from the CBD.
- MRI is considered the gold standard for initial evaluation and diagnosis of CCs.
- Endoscopic retrograde cholangiopancreatography and percutaneous transhepatic cholangiography are the most sensitive techniques; unfortunately, both are invasive and operator-dependent.
